# Investigating Carbon Coating on Ni-Invar and Ti–6Al–4V Surfaces for Low Friction Performance

**DOI:** 10.1021/acs.langmuir.5c01902

**Published:** 2025-08-08

**Authors:** Fatemeh Ghajari, Mobin Vandadi, Tabiri Kwayie Asumadu, Desmond Klenam, Winston Soboyejo, Nima Rahbar

**Affiliations:** † Department of Civil, Environmental and Architectural Engineering, 8718 Worcester Polytechnic Institute, Worcester, Massachusetts 01609-2280, United States; ‡ College of Engineering, 14627 State University of New York (SUNY) Polytechnic Institute, Utica, New York 13502, United States; § School of Chemical and Metallurgical Engineering, 37707 University of the Witwatersrand, Private Bag 3, WITS, Johannesburg 2050, South Africa

## Abstract

This study explored a sustainable method for depositing carbon-based coatings on Ni-Invar and Ti–6Al–4V metallic substrates using a pack carburization process with cyanide-rich *Manihot esculenta* (cassava) leaves as the carbon source. Comprehensive experimental and computational analyses were performed to investigate the composition, structure, and tribological performance of the coatings. Characterization using X-ray photoelectron spectroscopy (XPS), Raman spectroscopy, scanning electron microscopy (SEM), and lateral force microscopy (LFM) revealed that the Ni-Invar substrate developed turbostratic multilayer graphene coatings with minimal carbide formation, achieving ultralow coefficient of friction (COF) values of 0.08 (macroscale) and 0.0033 (nanoscale). In contrast, Ti–6Al–4V substrates formed coatings rich in titanium carbide and metal carbonates with more disordered carbon structures, resulting in higher COF values of 0.1 and 0.0147, respectively. Monte Carlo simulations illustrated an island growth mechanism on Ni-Invar driven by dominant carbon–carbon interactions, while Ti-6Al-4V exhibited uniform layer growth due to stronger carbon–substrate affinity. Density Functional Theory (DFT) calculations provided further insight, showing that Ti had a strong affinity for graphene, with a binding energy of −1.1440 × 10^3^ eV, whereas Ni and Fe exhibited stronger self-affinity (−1.6250 × 10^3^ and −1.7190 × 10^3^ eV, respectively), favoring metal–metal bonding over carbon–metal bonding. These integrated experimental and numerical analyses offer detailed insights into carbon coating structures and their growth mechanisms. The results underscore the critical role of substrate chemistry in determining coating morphology and tribological behavior, highlighting Ni-Invar as a promising candidate for achieving superlubricity through carbon-based surface engineering.

## Graphical abstract

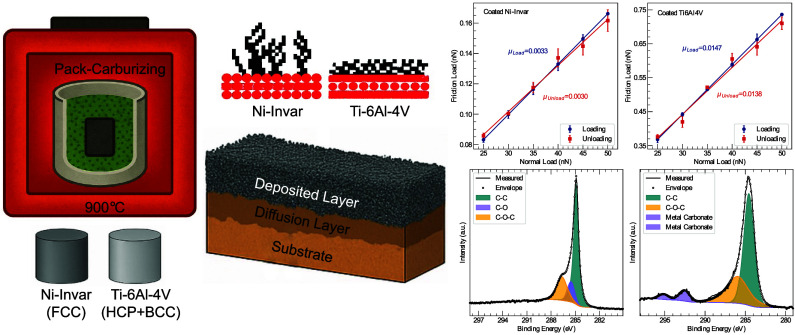

## Introduction

Historically, significant strides have been made not only in understanding the origins of friction but also in developing technologies to control it from the atomic to the macroscale. As a general technique, a small amount of lubricant applied to the contacting surfaces can significantly reduce friction and material loss.^1^ The significance of lubrication cannot be overstated, as nearly 23% of the world’s energy production is dissipated due to friction.^2^ Since the early 20th century, researchers have been exploring innovative methods to enhance the surface properties of various materials.^3^ During the past two decades, remarkable progress has been made in the design, development, and use of solid lubricant films.^4,5^ The current trend in modern tribology is to limit or reduce the use of liquid lubricants as much as possible (mainly due to environmental concerns) but increase the use of solid materials and coatings with self-lubricating properties.^6^ Generally, there are two main types of solid superlubricity: (1) structural superlubricity, realized in cases such as double-walled carbon nanotubes, graphene, and graphite; and (2) disordered solid interface-based superlubricity, demonstrated by DLC films.^7^

Furthermore, a wide range of metallic and nonmetallic additives, such as polymers, nanoparticles, and carbon nanomaterials, have been used to meet the growing demand for high-efficiency lubricants. Depending on the type and structure of the additives, different types of lubrication can be achieved. Due to the success and prevalence of graphite as a solid lubricant in industry for decades, carbon nanomaterials are one of the focal points of current tribological research. The large volume of research on carbon nanomaterials in tribology published between 2012 and 2022 highlights the significant interest in their tribological properties.^8,9^ Efforts to synthesize carbon-based structures, such as diamond and graphite, from low-pressure vapors date back to at least 1911.^10^ By the mid-1970s, Deryagin and Fedoseev reportedly achieved epitaxial growth of diamond films and whiskers through the pyrolysis of hydrocarbon–hydrogen gas mixtures.^11^ Following a decade of limited progress, the 1980s witnessed a surge in global interest in the synthesis and characterization of diamond films, an enthusiasm that continues today due to their remarkable physical and chemical properties.^12,13^

In this study, we focused on pack carburizing to enhance the surface tribological properties of two distinctly different metallic alloys, Ni-Invar and Ti–6Al–4V, with the aim of improving their surface friction.

One particularly promising method for depositing carbon-based materials is atmospheric pressure pack carburizing. This technique offers several advantages, including cost-effectiveness and procedural simplicity.

Researchers have demonstrated that the deposition of two-dimensional materials, such as graphene and MoS_2_, can lead to a superlubricious state on the surface.^14–17^ Superlubricity, characterized by an ultralow coefficient of friction of 10^–3^ or less, has been attributed to two primary mechanisms: (1) interlocking elimination (incommensurability) occurring at nano- to microscales,^18^ and (2) disordered mating interfaces governed by van der Waals forces, which mitigate stick–slip behavior.^19^ These factors have yielded coefficients of friction as low as 0.001 in experimental studies.

In this work, we used cyanide-rich powder derived from the leaves of *Manihot esculenta* (Cassava) as the carbon source and BaCO_3_ as the activator. This special plant was selected due to its abundant carbon content, which facilitates the formation of coatings under high-temperature conditions.^20^ At elevated temperatures, the triple bond of carbon to nitrogen atoms dissociates, enabling the deposition of a thin layer through carburizing processes.

Building on this sustainable feedstock, this study advances the field in three key aspects. Graphene-like carbon coatings are produced from a low-cost cassava precursor via atmospheric pressure pack carburizing, demonstrating a scalable and economically attractive route suitable for industrial deployment. Substrate choice is shown to dictate the growth mode: Ni-Invar promotes Volmer–Weber island formation, whereas Ti–6Al–4V favors Frank–van der Merwe layer-by-layer deposition, thereby enabling morphology control without altering the deposition chemistry. Coupling atomistic Monte Carlo simulations with *ab initio* density functional theory (DFT) establishes a quantitative correlation between carbon–metal and carbon–carbon interaction energies (Δ*E*_C–M_ versus Δ*E*_C‑C_) and the resulting architectures, providing a predictive framework for rational coating design. Collectively, these advances lay the foundation for the comprehensive experimental and computational characterization presented in the following sections.

To characterize and analyze the resulting coatings, we employed a combination of experimental techniques, including X-ray photoelectron spectroscopy (XPS), Raman spectroscopy, and electron microscopy. These experiments were complemented by lateral force microscopy (LFM) of the coated samples to evaluate the impact on the coefficient of friction. In addition, atomic Monte Carlo simulations and density functional theory (DFT) were utilized to investigate the structural and electronic properties of the deposited layers. This integrated approach enabled us to comprehensively assess the composition, morphology, and functional performance of the coatings on the Ni-Invar and Ti–6Al–4V surfaces, providing valuable insights into the mechanisms governing their formation and tribological behavior.

## Methods

### Alloys and Coating Process

In this study, two different substrates, Ni-Invar and Ti–6Al–4V, were used for coating. Invar alloy consists of 64% Fe and 36% Ni, exhibiting a face-centered cubic (FCC) structure, while Ti–6Al–4V comprises 90% Ti, 6% Al, and 4% V, with a combination of hexagonal close-packed (HCP, α-phase) and body-centered cubic (BCC, β-phase) structures. Cylindrical samples with a diameter of 2.54 cm and a height of 2 cm were cut and polished using a Buehler EcoMet 30 semiautomatic grinder-polisher to achieve a surface roughness below 1 μm.

The deposition process was carried out by pack carburizing, employing a mixture of powdered dried *Manihot esculenta* (cassava) leaves and BaCO_3_ as the carburizing energizer in a 1:3 ratio. The alloy cylinders were immersed in a ZrO crucible containing the powder mix and heated to 900 °C for 6 h to facilitate the carburization process.

### X-ray Photoelectron Spectroscopy

To investigate the deposited layer on the surface of the alloys, X-ray photoelectron spectroscopy (XPS) was employed. A Thermo Fisher Scientific Nexsa XPS system, equipped with a 400 μm X-ray gun, was used for the analysis. The test was conducted over an area of 1 × 1 mm^2^, with measurements taken at 9 scanned points to mitigate any potential effects of X-ray exposure on subsequent points. The collected data were analyzed by using Avantage software, enabling precise characterization of the surface composition and chemical states within the deposited layers.

### Raman Spectroscopy

Raman spectroscopy was employed to confirm the formation of a graphitic structure on the sample surfaces.^21^ A Horiba XploRa Raman Micro Spectrometer with a 532 nm laser beam was used for the analysis. Calibration of the instrument was performed by using a silicon wafer with a (100) plane orientation and the characteristic 520 cm^–1^ peak. Data normalization and baseline correction were carried out using LabSpec 6 software to ensure accurate and reliable results.

### Lateral Force Microscopy

Lateral force microscopy (LFM) was performed using a Park Systems NX20 atomic force microscopy (AFM). Measurements were conducted over a 5 × 5 μm^2^ scan area, and the results were averaged to determine the coefficient of friction for each sample. The applied normal load was varied from 25 to 50 nN in 5 nN increments. A Park Systems PPP-CONTSCR cantilever was used.

### Ball-on-Disk Wear Test

Ball-on-disk wear tests were conducted by using an Anton Paar TRB^3^ tribometer within a humidity-controlled chamber. The relative humidity was varied from 10% to 80% in 10% increments to assess its influence on the performance of the deposited layer. The results showed that variations in the coefficient of friction remained within 5% across the tested humidity range. Accordingly, the data presented in the following sections correspond to the tests performed at 50% relative humidity.

Experiments were conducted on both treated and untreated substrates under a constant normal load of 2 N. The counter bodies consisted of 6 mm diameter 100Cr6 steel balls, which were either uncoated or coated using the same process applied to the alloy substrates. The angular velocity was maintained at 100 rpm, and the friction data were recorded at a sampling rate of 20 Hz.

Each test was conducted over a fixed sliding distance of 100 m to ensure comparability of wear rates across different sample conditions. Prior to testing, all samples and counter bodies were ultrasonically cleaned in ethanol to remove surface contaminants that could affect the tribological performance. After testing, the wear track on the disk and the wear scar on the ball were analyzed by using optical profilometry and scanning electron microscopy (SEM) to evaluate wear mechanisms and quantify material loss. To ensure accurate humidity control, the environmental parameters were allowed to stabilize for 30 min before each test. All measurements were carried out at room temperature (22 ± 1 °C) to minimize the influence of thermal variations on the tribological response.

### Electron Microscopy

To investigate the morphology of the deposited layer, scanning electron microscopy (SEM) was employed by using a Thermo Scientific Scios 2 DualBeam system. Morphological images were acquired using secondary electron (SE) detection, while compositional contrast was obtained by using backscattered electron (BSE) imaging. SEM images were captured at an accelerating voltage of 10 kV, and energy-dispersive spectroscopy (EDS) maps were collected using a 20 kV beam, a dwell time of 5 μs, and a beam current of 0.8 nA. Prior to imaging, all samples were coated with a thin conductive gold layer using a sputter coater to minimize charging effects. Multiple regions were examined for each sample to ensure that the observed features were representative of the overall surface. The image contrast and resolution were optimized to reveal key surface characteristics, such as grain boundaries, voids, and coating uniformity.

### Atomic Monte Carlo Simulation

To better understand the deposition process and compare the structures of the deposited layers on different substrates, a Monte Carlo (MC) simulation was run in Python. For this purpose, an initial 2D system was created, and a group of atoms with the crystal structure of the corresponding substrate was assumed at the bottom (001 FCC for Ni-Invar and 0001 HCP for Ti–6Al–4V). The atoms are inserted into the system from the top plane at random locations and random angles. A periodic boundary condition was assumed for the side planes. In the case of contact between the new atom and a previous atom (substrate or already deposited atoms), two equations were used. (i) Reflection probability, which is a probability function calculating the chance of the new particle being reflected back based on the angle of attack, and was calculated using^22^

1 $$P_{\mathrm{reflection}}=P_{0}+\Delta P\mathrm{si}n^{2}\left(\theta \right)$$

where *P*_0_ is the base probability of deposition at angle zero, and Δ*P* is the change in probability as the angle increases, setting the maximum probability of deposition at 90 deg. (ii) Surface adhesion probability, which is the probability of deposition based on the energy change between the two atoms. For this, density functional theory simulations were performed to measure the energy change of carbon atoms near carbon, titanium, and nickel atoms. Afterward, two probability terms were calculated for each system, $\frac{\Delta E_{C‐C}}{\Delta E_{C‐C}+\Delta E_{C‐\mathrm{Ni}}}$ and $\frac{\Delta E_{C‐C}}{\Delta E_{C‐C}+\Delta E_{C‐\mathrm{Ti}}}$ . Based on these two probabilities, the deposition was modeled.

### Density Functional Theory Simulation

To assess the likelihood of metal carbide formation on the surface during treatment, Density Functional Theory (DFT) simulations were performed using the Generalized Gradient Approximation (GGA) to evaluate changes in the system energy under various bonding configurations. Specifically, the binding energies of the Ni and Fe atoms were calculated when they were bonded to the Invar alloy and when they interacted with graphene.

To simplify the complex structure of Invar, it was approximated by using three representative phases: Fe_3_Ni, FeNi, and FeNi_3_.^23^ For the Ti–6Al–4V alloy, the system was modeled using only titanium atoms due to the predominance of Ti relative to Al and V in determining the bonding behavior. As Ti–6Al–4V exhibits both body-centered cubic (BCC) and hexagonal close-packed (HCP) phases at 900 °C, the binding energy of a titanium atom was evaluated in three configurations: bonded to graphene, to BCC titanium, and to HCP titanium.^24^

To simulate these interactions, the initial system was constructed with a single atom positioned at varying distances from the substrate surface, ranging from 5 to 1.5 Å. *Ab initio* molecular dynamics (AIMD) simulations were carried out at 900 °C. The total energy of the system was averaged over a 50 ps trajectory with a 1 fs time step, following an initial equilibration period of 5 ps.

## Results and Discussion

### Wear and Friction of Coated Ni-Invar and Ti–6Al–4V

The results of the ball-on-disk wear test (Figure 1a) indicate that following the initial running-in phase, the coefficient of friction (COF) for the Ni-Invar-coated sample is approximately an order of magnitude lower than that of the Ti–6Al–4V sample. Specifically, the COF for the coated Ni-Invar sample decreased significantly from 0.7 to 0.08. In contrast, the reduction in the Ti–6Al–4V sample was less pronounced (Figure 1b), with the COF decreasing from 0.6 to 0.1. For both samples, the tests were carried out over a maximum of 20,000 cycles, with the results presented using a scaled cycle axis to facilitate comparison.

**Figure 1:**
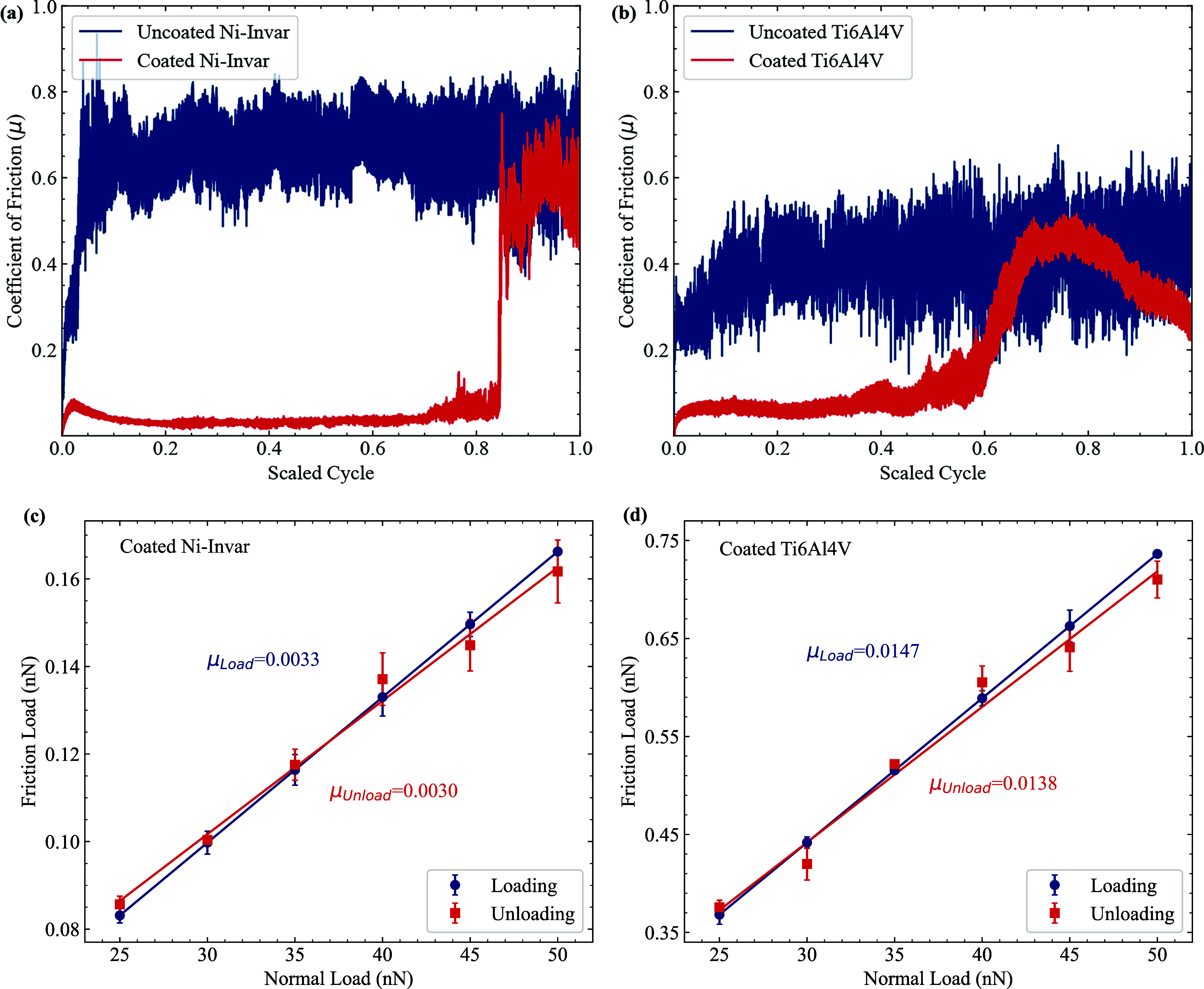
(a) and (b) show the coefficients of friction for the coated and uncoated Ni-Invar and Ti–6Al–4V samples, respectively, using a ball-on-disk tribometer at a rotation rate of 100 rpm. Following the run-in period, the steady-state coefficient of friction for the coated Ti–6Al–4V is 0.1, and that for the coated Ni-Invar is 0.08. The *x*-axis represents the scaled cycle number, defined as the actual number of cycles divided by the maximum number of cycles (20,000 for both samples), enabling comparison between samples. (c) and (d) present frictional force measurements for coated Ni-Invar and Ti–6Al–4V samples, respectively, obtained via lateral force microscopy (LFM) at a scan rate of 0.05 μm/s. The measured coefficients of friction are 0.0033 for Ni-Invar and 0.0147 for Ti–6Al–4V.

The difference in the COF measured by the LFM versus the ball-on-disk method arises from their distinct operational scales; the tribometer operates at the macroscale, where measurements are influenced by surface roughness, material deformation, and asperity interactions. The LFM operates on the microscale and focuses on adhesive and van der Waals interactions, resulting in lower COFs. Furthermore, the normal loads in the tribometer are much higher than the loads applied in LFM.^25^

Lateral force microscopy (LFM) was performed after deposition to measure the frictional force on the 1 μm × 1 μm coating applied to each substrate (Figure 1c,d). The test was conducted under varying normal loads: the load was first increased from 25 to 50 nN and then decreased back to 25 nN in order to assess the effect of higher loads on the coefficient of friction and the structural integrity of the coating layers. The results demonstrate that the deposited coatings can withstand the applied loads without mechanical failure. As shown in Figure 1c,1d, there is a positive linear relationship between the friction force (*F*_f_) and the normal load (*F*_N_) in both the loading and unloading regimes. The slope of the linear fit of *F*_f_–*F*_N_ defines the coefficient of friction. For the Ni-Invar sample, the average coefficient of friction was approximately 0.0033, while for the Ti–6Al–4V sample, it averaged around 0.0147.

### Characterization of the Deposited Layer

To understand the difference in friction coefficients between the two alloys, as well as the reduction in friction compared to the uncoated samples, the coated surfaces were characterized using X-ray photoelectron spectroscopy (XPS) and Raman spectroscopy. XPS analysis provides information about the bonding environment and elemental composition of the deposited layers. As shown in Figure 2, the carbon spectra of both materials exhibit a strong sp^2^ C signal, confirming the formation of graphitic structures on the surface. This observation is consistent with the characteristic features identified in the Raman spectra. Moreover, the pronounced intensity of the C–O bonds indicates substantial oxygen incorporation into the deposited carbon, which likely contributes to the observed shifts in the Raman peak positions and enhanced G peak intensity. The absence of nitrogen peaks (supplementary figures) suggests that nitrogen atoms released from cyanide precursors are not incorporated into the deposited layers.

**Figure 2:**
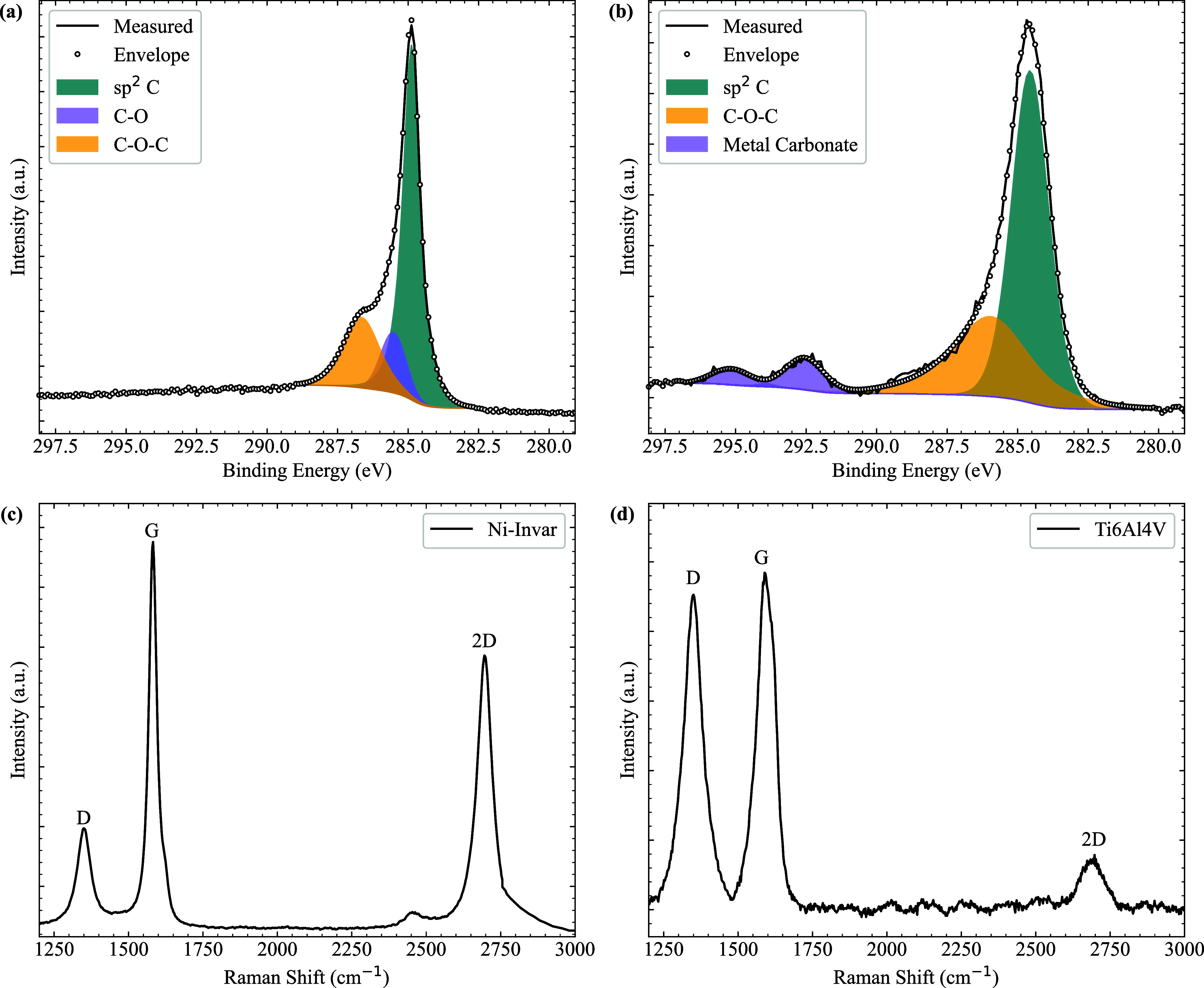
X-ray photoelectron spectroscopy (XPS) and Raman spectroscopy of the coated Ni-Invar and Ti–6Al–4V samples. (a) The C 1s spectrum of Ni-Invar shows the presence of sp^2^-hybridized carbon, C–O, and C–O–C bonding on the surface. (b) The C 1s spectrum of Ti–6Al–4V reveals the formation of metal carbonates, in addition to sp^2^ carbon and C–O–C bonding. (c) and (d) Raman spectra of the coated Ni-Invar and Ti–6Al–4V samples, respectively, displaying the characteristic graphene features on both surfaces.

For the Ni-Invar substrate, the absence of metal carbide formation can be attributed to the phase stability of the alloy at the treatment temperature. At 900 °C, iron exists in its austenitic phase (γ-Fe), and the presence of nickel, an austenite stabilizer, suppresses the formation of iron carbides during cooling. This thermodynamic effect inhibits the reaction between carbon and iron, thereby preventing the formation of Fe-carbide phases and resulting in a predominantly carbonaceous surface rather than a carbide-rich layer. As a result, no metal carbide signatures were detected in the XPS spectra of the Ni-Invar sample. In contrast, the Ti–6Al–4V substrate exhibits stronger chemical interactions between Ti/Al and carbon at the same temperature, promoting the formation of carbide and carbonate species, as evidenced by the appearance of metal carbonate peaks in its XPS spectra.

The Raman spectra further confirm the presence of graphitic structures in the coatings, though with notable differences between the two substrates. The spectral characteristics of the Ni-Invar and Ti–6Al–4V coatings show variations in their ratios *I*_D_/*I*_G_ and *I_2D_*/*I*_G_, reflecting differences in the disorder and stacking characteristics.^26^ For the Ni-Invar sample, the *I*_D_/*I*_G_ ratio is relatively low, approximately 1/3, indicating moderate disorder consistent with multilayer graphene containing defects, likely at grain boundaries or edges. The higher *I*_2D_/*I*_G_ ratio suggests the presence of turbostratic multilayer graphene with relatively ordered stacking. In contrast, the Ti–6Al–4V sample exhibits a significantly higher *I*_D_/*I*_G_ ratio, approaching 1, indicating a substantially higher degree of disorder and structural defects. This suggests that the carbon layer on Ti–6Al–4V is less graphitic. Furthermore, the lower *I*_2*D*_/*I*_G_ ratio in Ti–6Al–4V compared to that in Ni-Invar confirms the lack of well-ordered graphene layers and the presence of substantial defects, likely due to stronger interactions between the deposited carbon atoms and the metallic substrate. These differences in Raman characteristics align with the tribological performance of the coatings, where the more ordered graphene layers on Ni-Invar contribute to lower friction, while the defective carbon structure on Ti–6Al–4V results in a higher friction coefficient.

Comparing the *I*_D_ intensity of the coated samples to that of SP1 grade graphite, more pronounced D peaks were observed, indicating the presence of carbon oxide structures.^26^ The elevated *I*_D_/*I*_G_ ratio observed in the Ti–6Al–4V sample suggests that oxidation introduces a significant fraction of *sp* ^3^-hybridized carbon atoms, disrupting the pristine graphene lattice through the incorporation of oxygen-containing functional groups. Furthermore, the substantial attenuation of the 2*D* peak in the Ti–6Al–4V sample implies that oxidation and substrate interactions significantly degrade the long-range order of the π-conjugated system. These observations collectively indicate that the carbon layer formed on Ti–6Al–4V is more structurally disordered than that formed on the Ni-Invar substrate.

### Film Growth Progression

To analyze the topology and morphology of the deposited layer, scanning electron microscopy (SEM) was performed on the coated samples. Figure 3 presents the surface morphologies of the Ti–6Al–4V and Ni-Invar substrates after 6 h of treatment. The SEM images reveal that the deposited layer on Ni-Invar exhibits a bushy morphology, whereas on Ti–6Al–4V, the layer adopts a rod-like structure. To further investigate the underlying reasons for these morphological differences, the deposition was intercepted after 3 h, and the intermediate stages of film growth were examined.

**Figure 3:**
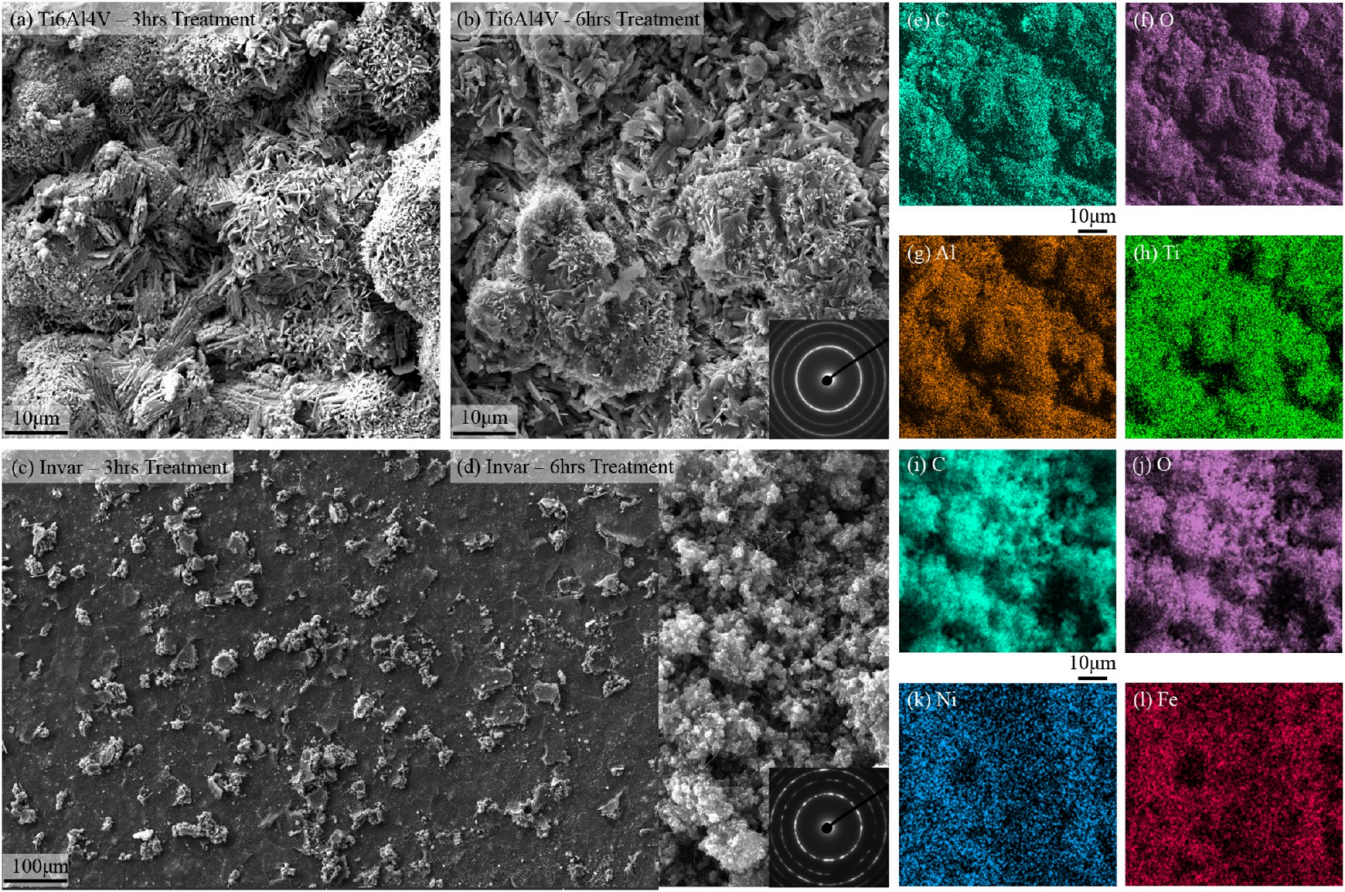
Scanning electron microscopy (SEM) images and energy-dispersive spectroscopy (EDS) elemental maps of Ni-Invar and Ti–6Al–4V samples treated for 3 and 6 h. (a) SEM image of Ti–6Al–4V after 3 h of treatment showing early-stage surface coverage and film growth. (b) SEM image of TTi–6Al–4V after 6 h showing continued film development with a rod-like morphology; the inset displays a selected area electron diffraction (SAED) pattern. (c) SEM image of Ni-Invar after 3 h showing the formation of bushy clusters, primarily along the grain boundaries. (d) SEM image of Ni-Invar after 6 h showing that these clusters coalesce to form a continuous film over the surface; the inset shows the corresponding SAED pattern. (e–h) EDS elemental maps of the Ti–6Al–4V sample treated for 6 h showing the distributions of (e) C, (f) O, (g) Al, and (h) Ti, confirming the uniform distribution of carbon. (i–l) EDS elemental maps of the Ni-Invar sample treated for 6 h showing the distributions of (i) C, (j) O, (k) Ni, and (l) Fe, further highlighting the uniform carbon coverage across the coated surfaces.

SEM images of the samples treated for 3 h reveal that on the Ni-Invar substrate, the deposited atoms primarily form dispersed clusters rather than a continuous film. This clustering behavior can be attributed to thermodynamic factors: the deposited atoms favor aggregation over bonding with the substrate due to the lower energy state associated with cluster formation. In contrast, the Ti–6Al–4V sample exhibits an almost uniformly coated surface after the same treatment duration. This suggests that in this system, the interaction energy between the deposited atoms and the substrate is more favorable than the cohesive energy among the deposited atoms, thereby promoting continuous film growth instead of cluster formation.

Furthermore, backscattered electron SEM images reveal that, in the case of Ni-Invar, the initial clusters form predominantly along grain boundaries. This is attributed to the higher atomic energy at the grain boundaries, which makes them more favorable sites for nucleation. In contrast, the atoms within the grains remain more energetically stable, leading to preferential deposition along the grain boundaries rather than within the grains themselves.

The SEM observations shown in Figure 4 indicate that after 3 h of treatment, the Ti–6Al–4V substrate develops a uniform, continuous film, while the Ni-Invar substrate exhibits discrete carbon clusters. Assuming equal availability of carbon atoms at the surfaces of both samples, these contrasting morphologies can be attributed to differences in the binding energies between the carbon atoms and the respective substrate materials.

**Figure 4:**
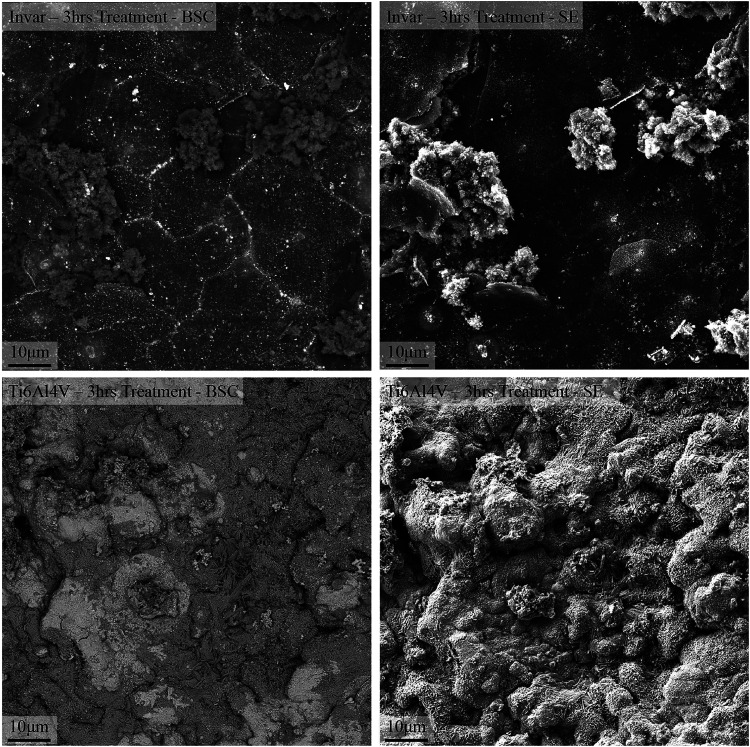
Secondary electron and backscattered electron microscopy images of the Ni-Invar and Ti–6Al–4V samples. The Ti–6Al–4V sample exhibits a layer-by-layer growth mode, resulting in uniform and full surface coverage from the early stages of deposition. In contrast, the Ni-Invar sample demonstrates an island growth mechanism, where carbon is initially deposited along the grain boundaries, and the resulting clusters gradually expand and coalesce to form a continuous coating.

Atomistic Monte Carlo simulations suggest that the cluster formation of carbon atoms on the Ni-Invar surface can be attributed to the difference in the interaction energies: Δ*E*_C‑C_, Δ*E*_C‑M_, and Δ*E*_M‑M_, where M represents either Ni or Fe. As shown in Figure 5, the deposited atoms initially form isolated clusters on the Ni-Invar surface rather than spreading uniformly. Over time, these clusters expand and eventually coalesce, but the surface remains partially exposed, indicating that the carbon–carbon interaction energy (Δ*E*_C‑C_) dominates that of the carbon–metal interactions, favoring agglomeration rather than continuous film growth. This is consistent with the results from XPS.

**Figure 5:**
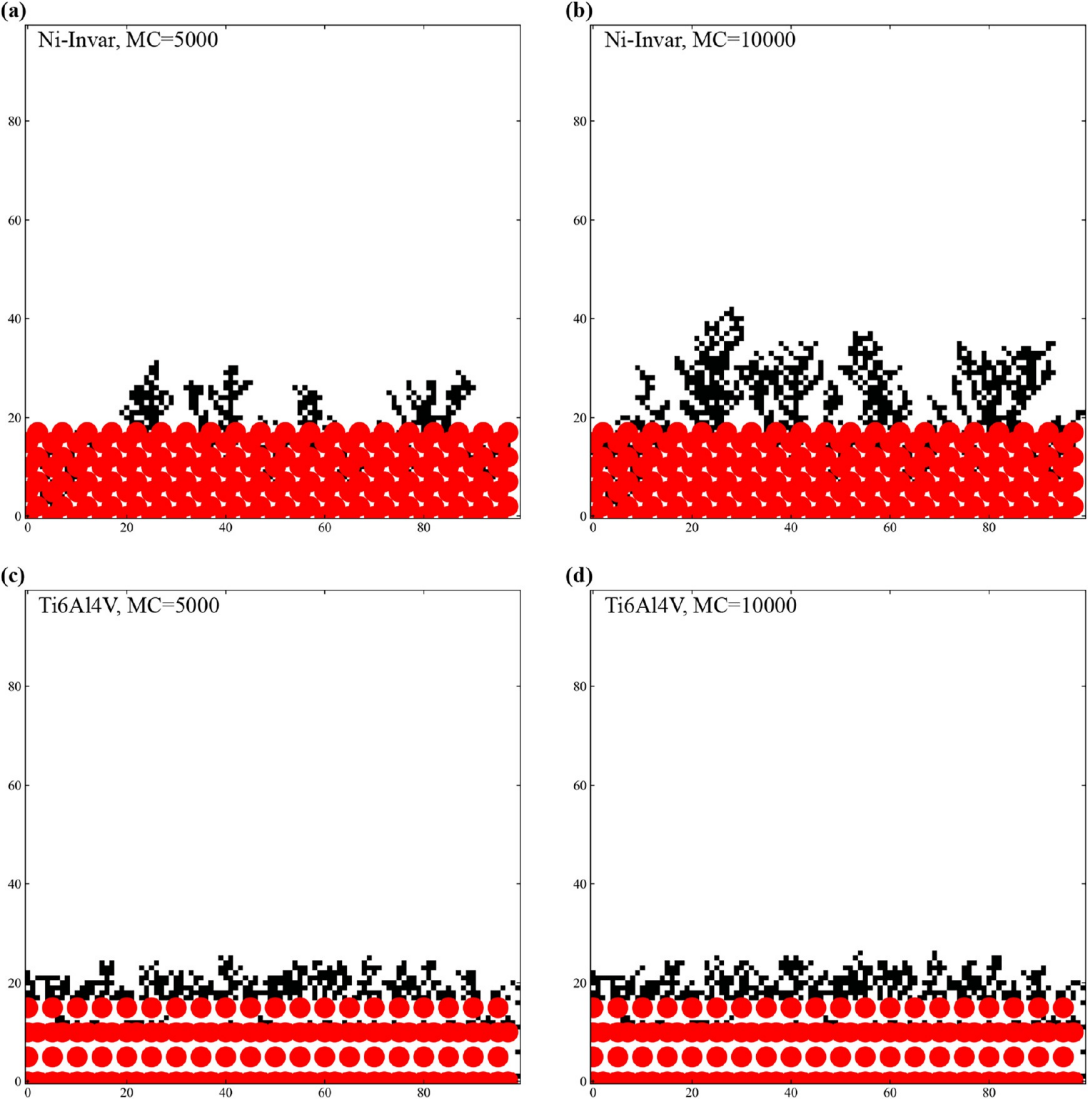
Monte Carlo simulation results for Ni-Invar (a, b) and Ti–6Al–4V (c, d) substrates. Black dots represent deposited carbon atoms, while red circles indicate metallic substrate atoms. (a, b) Island growth mode on the Ni-Invar substrate, attributed to the low affinity of Ni for bonding with carbon as well as its inhibitory effect on Fe–C bonding. In contrast, (c) and (d) display a layer-by-layer growth mode on the Ti–6Al–4V substrate. Following the initial surface coverage, a random forest-like growth pattern develops, resembling the behavior commonly observed in physical vapor deposition processes.

In contrast, the Monte Carlo simulation for Ti–6Al–4V, which considers the interaction energies Δ*E*_C‑C_, Δ*E*_C‑Ti_, and Δ*E*_Ti‑Ti_, reveals a distinctly different growth mechanism. Initially, carbon atoms uniformly cover the Ti substrate, indicating that the carbon–titanium interaction (Δ*E*_C‑Ti_) is stronger than the carbon–carbon interaction. This strong affinity promotes layer-by-layer deposition, effectively suppressing cluster formation and producing a uniform coating. As the deposition continues, the growth remains homogeneous, forming a thicker layer in which the substrate’s influence progressively diminishes.

These simulation results are consistent with the experimental observations, which demonstrate localized carbon cluster formation on Ni-Invar but uniform film deposition on Ti–6Al–4V. The different morphologies observed are fundamentally governed by differences in the energy of carbon–substrate interaction, which ultimately affects the structural and tribological properties of the coatings.^27,28^

In Monte Carlo deposition simulations, four primary interaction mechanisms between the substrate and incident atoms are typically considered: (i) reflection, in which the incident atom rebounds from the substrate surface; (ii) resputtering, where the incoming atom transfers sufficient energy to displace a previously deposited atom, causing the ejection of the deposited atom and its replacement by the incident one; (iii) biased diffusion, where the deposited atom migrates across the surface before settling into a stable position; and (iv) athermal diffusion, in which the kinetic energy from the deposited atom rearranges already deposited atoms, altering the film structure. In this study, only reflection and resputtering mechanisms were included, as the probabilities of biased and athermal diffusion are low enough to justify their exclusion.^22,29^

Moreover, temperature significantly influences Monte Carlo simulations since the kinetic energy of incident atoms affects the reflection and resputtering probabilities. However, in the current work, the temperature effect was simplified into a probabilistic factor that governs the deposition process. Although this simplification introduces some deviation from the experimental data, the magnitude of the resulting error is expected to be minimal, as the temperature-to-melting temperature ratio (*T*/*T*_m_) exceeds 0.5 for both substrates.^22^

Figure 6 presents the interaction energy profiles for nickel (left) and titanium (right) atoms as they approach different metallic substrates and graphene. For Ni-Invar, a solid solution without a fixed stoichiometry and three possible surface compositions (Fe_3_Ni, FeNi, and FeNi_3_) were selected. Both Ni and Fe atoms were individually brought toward each alloy surface as well as graphene to evaluate their relative stability. For the Ti–6Al–4V system, substrates with two distinct crystal structures, hexagonal close-packed (HCP) and body-centered cubic (BCC), were considered, and a Ti atom was moved similarly toward each substrate and graphene.

**Figure 6:**
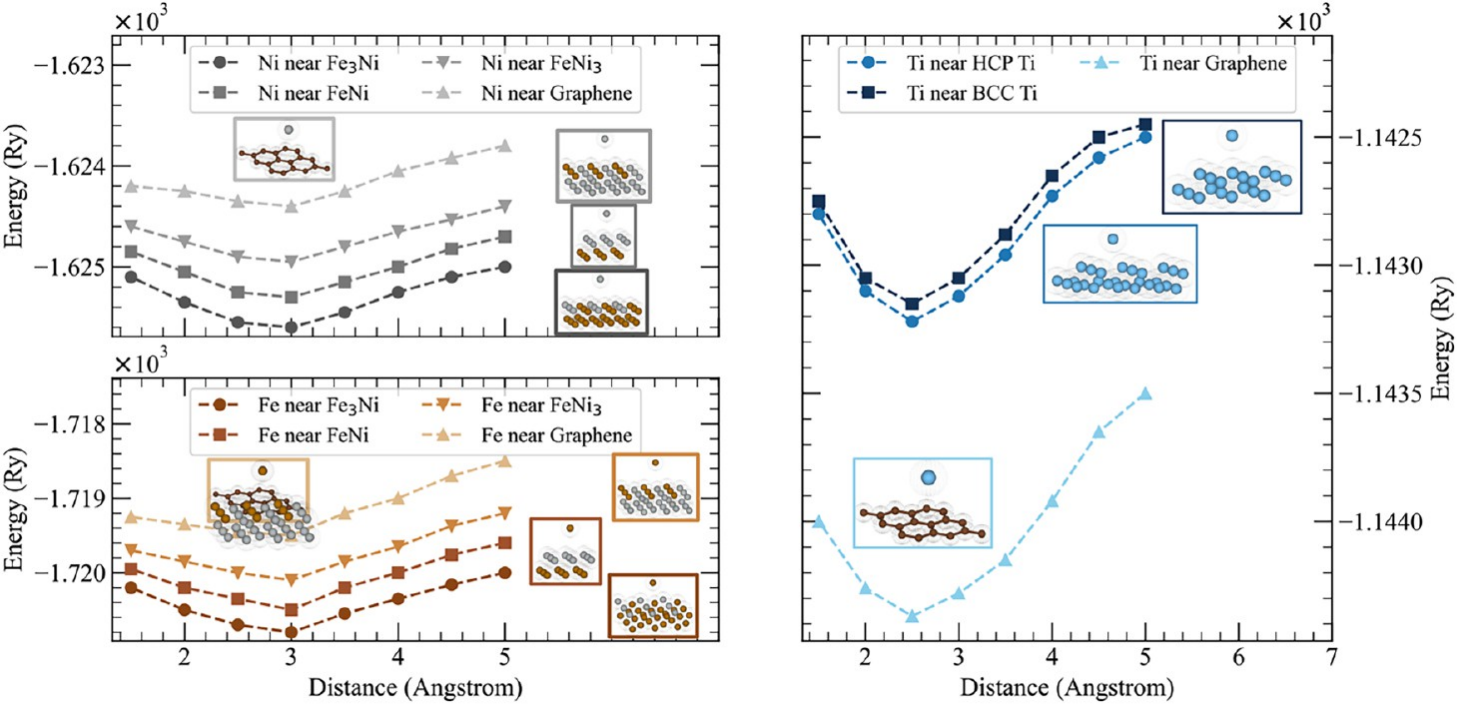
DFT-calculated energy as a function of interatomic distance for Ni, Fe, and Ti atoms interacting with various substrates. The top-left subplot (gray shades) presents the interactions between Ni atoms and different Ni-Invar compositions (Fe_3_Ni, FeNi, and FeNi_3_) as well as graphene. The bottom-left subplot (brown shades) shows similar interactions for the Fe atoms. The right subplot (blue shades) illustrates the interaction of Ti atoms with the HCP Ti, BCC Ti, and graphene substrates. The insets display the atomic configurations corresponding to each interaction type, aligned with their respective energy profiles. The left-side plots indicate that the Ni and Fe atoms exhibit stronger binding to the Ni-Invar substrates than to the graphitic layer. In contrast, the right-side plot demonstrates that Ti atoms have a more favorable (lower energy) interaction with the graphitic layer compared to both the HCP and BCC titanium substrates.

In the titanium system, strong metallic bonding dominated by *d*-band interactions is observed when a Ti atom approaches an HCP Ti surface, with the system energy becoming significantly more negative around 2.5–3.0 Å. At distances shorter than approximately 2.0 Å, however, the energy increases due to Pauli repulsion and electron cloud overlap. Ti–graphene interactions, on the other hand, involve d−π hybridization, electron transfer, and van der Waals forces, which collectively result in stronger overall binding compared to that of pure Ti-Ti interactions. Partial electron donation from Ti to the graphene π-system further stabilizes the atom, although the equilibrium binding distance is somewhat larger (approximately 2.2–3.0 Å) due to the semicovalent nature of this interaction. These observations suggest that titanium atoms exhibit a stronger preference for binding with graphene than with metallic titanium surfaces, consistent with the XPS evidence of metallic carbide formation at the surface.

For nickel, which adopts a face-centered-cubic (FCC) structure, similarly strong metallic bonding arises due to the delocalized *d*-electrons. As a Ni atom approaches an FCC Ni surface, the system energy decreases markedly around 2.5–3.0 Å. At closer distances (<2.0 Å), the energy increases due to Pauli repulsion. In contrast, the Ni–graphene interactions are comparatively weaker and predominantly governed by van der Waals forces rather than significant hybridization with the graphene π-system. As a result, Ni atoms consistently favor metallic bonding with Ni-based surfaces over adsorption onto graphene.

Likewise, Fe atoms that approach Ni-Invar substrates (Fe_3_Ni, FeNi, and FeNi_3_) exhibit strong metallic Fe–Fe interactions, characterized by a substantial energy reduction at typical metallic bonding distances (approximately 2.5–3.0 Å). In contrast, Fe–graphene interactions are mainly governed by weaker van der Waals forces, resulting in relatively higher (less negative) energies and, consequently, less favorable adsorption. Thus, similar to Ni, Fe atoms preferentially bind to metallic substrates rather than to graphene surfaces.

## Conclusions

This study demonstrates that the morphology and tribological performance of carbon coatings formed via pack carburizing are strongly influenced by the substrate’s chemical characteristics. On Ni-Invar, the coating developed into turbostratic multilayer graphene with minimal carbide formation, achieving a low COF of 0.08 at the macroscale and 0.0033 at the nanoscale. In contrast, Ti–6Al–4V promoted the formation of disordered carbide-rich carbon layers, resulting in higher friction values.

Monte Carlo and density functional theory (DFT) simulations revealed that the weaker carbon–substrate interactions in Ni-Invar promote carbon clustering and the formation of incommensurate interfaces, conditions favorable for achieving superlubricity. Conversely, the stronger carbon–metal affinity in Ti–6Al–4V encourages uniform film growth and carbide formation, which disrupts the graphitic order and increases friction.

These findings highlight the pivotal role of atomic-scale interactions in determining the coating architecture and tribological behavior. The demonstrated ability to engineer ultralow-friction surfaces using sustainable carbon sources indicates that Ni-Invar is a promising candidate for advanced applications in energy-efficient systems, including microelectromechanical systems (MEMS), aerospace bearings, and precision instrumentation components.

## Supplementary Material



## Notes

XPS spectra of oxygen, nitrogen, iron, and nickel elements in the coated layer of Ni-Invar (Figure S-1); XPS spectra of oxygen, nitrogen, titanium, and aluminum elements in the coated layer of Ti–6Al–4V (Figure S-2) (PDF)
