# Supplementary material for: Investigating Carbon Coating on Ni-Invar and Ti–6Al–4V Surfaces for Low Friction Performance
Source: Langmuir. 2025 Aug 8;41(32):21425–34. doi: 10.1021/acs.langmuir.5c01902 (PMC12368999; doi:10.1021/acs.langmuir.5c01902)
Supplement: Supplementary file 1 [file la5c01902_si_001.pdf]

Supplementary Information for Investigating Carbon Coating on Ni-Invar and Ti-6Al-4V Surfaces for Low Friction Performance

Fatemeh Ghajari[1], Mobin Vandadi[1], Tabiri Asumadu[2], Desmond Klenam[3], Winston Soboyejo[2], and Nima Rahbar[1*]

[1]Department of Civil, Environmental and Architectural Engineering, Worcester Polytechnic Institute, Worcester, MA, USA
[2]College of Engineering, State University of New York (SUNY) Polytechnic Institute, Utica, NY, United States
[3]School of Chemical and Metallurgical Engineering, University of the Witwatersrand, Private Bag 3, WITS, 2050, South Africa
[*]Corresponding author. Email: nrahbar@wpi.edu

Below are the XPS spectra of additional elements present in the Ni-Invar and Ti-6Al-4V alloys. For the Ni-Invar alloy, the signal-to-noise ratios (SNR) for nickel, iron, and nitrogen are sufficiently low that their corresponding signals can be considered negligible for conclusive analysis. Thus, it can be deduced that the coated layer on the Ni-Invar consists of only carbon and oxygen atoms.

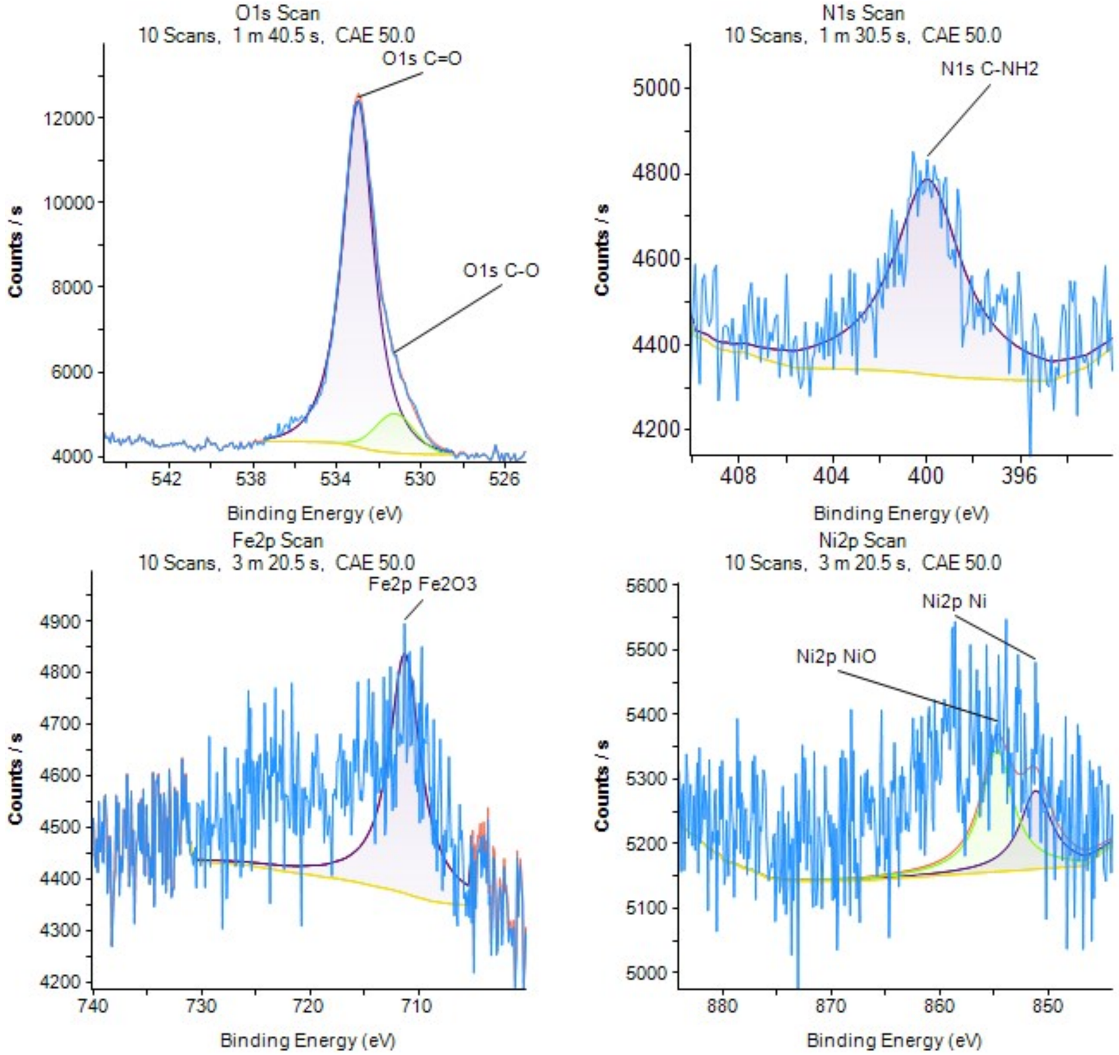

Figure 1: XPS spectra of Oxygen, Nitrogen, Iron and Nickel elements in the coated layer of Ni-Invar. The results for the N, Fe, and Ni are unreliable due to their high signal-to-noise ratio.

In the case of the Ti-6Al-4V alloy, the SNR for nitrogen remains low, suggesting that the incorporation of nitrogen atoms into the coated layer is inconsistent or limited within the structure. In contrast, the SNRs for titanium and aluminum are sufficiently high, indicating presence of these elements in the caoted layer, which in turn influences the structural integrity of the coating.

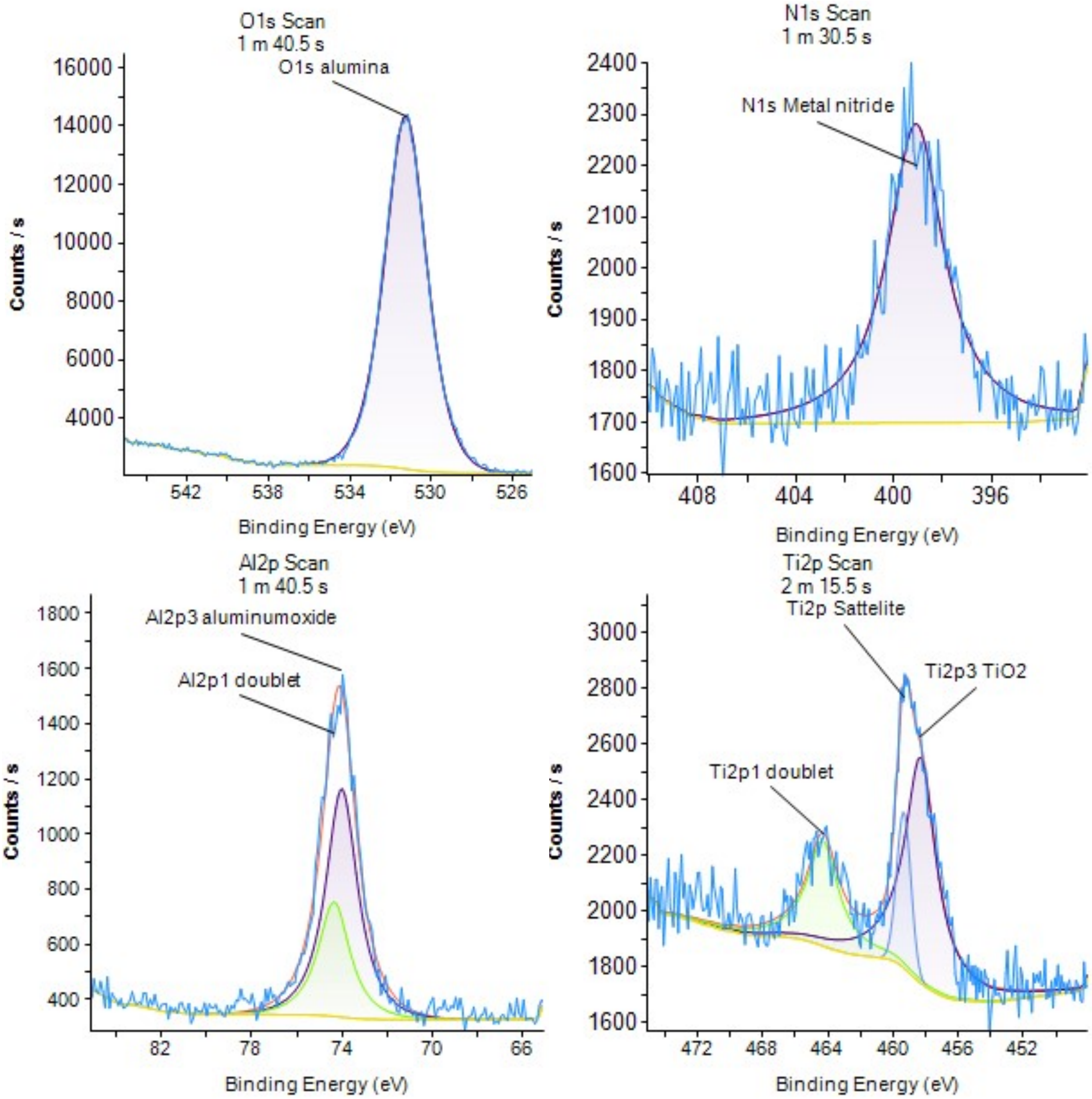

Figure 2: XPS spectra of Oxygen, Nitrogen, Titanium and Aluminum elements in the coated layer of Ti-6Al-4V. The peaks for the Ti and Al are distinct and shows presence in the coated layer, which is consistent with the energy interaction of these elements and carbon.
